# Pre-birth acquisition of personhood: Incremental accrual of attributes as the framework for individualization by serial and concurrently acting developmental factors

**DOI:** 10.3389/frph.2023.1112935

**Published:** 2023-03-20

**Authors:** Claude L. Hughes, Gavin C. Hughes

**Affiliations:** ^1^ Department of Obstetrics and Gynecology, Duke University Medical Center and Therapeutic Science and Strategy Unit, IQVIA, Durham, NC, United States; ^2^ Departments of Philosophy and Biology, UNC Neuroscience Center and the BRAIN Initiative Viral Vector Core, University of North Carolina at Chapel Hill, Chapel Hill, NC, United States

**Keywords:** personhood, epigenetics, biological ogranismic clock, physiogenesis, potential human, future human, prenatal developmental factors

## Abstract

Discrete events and processes influence development of individual humans. Attribution of personhood to any individual human being cannot be disconnected from the underlying biological events and processes of early human development. Nonetheless, the philosophical, sociological and legal components that are integral to the meaning of the term as commonly used cannot be deduced from biology alone. The challenge for biomedical scientists to inform discussion in this arena then rests on profiling the key biological events and processes that must be assessed when considering how one might objectively reason about the task of superimposing the concept of personhood onto the developing biological entity of a potential human being. Endogenous genetic and epigenetic events and exogenous developmental *milieu* processes diversify developmental trajectories of potential individual humans prior to livebirth. First, fertilization and epigenetic resetting of each individual's organismic clock to time zero (*t* = 0) at the gastrulation/primitive streak stage (day 15 of embryogenesis), are two discrete unseen biological events that impact a potential individual human's attributes. Second, those two discrete unseen biological events are immersed in the continuous developmental process spanning pre-fertilization and gestation, further driving individualization of diverse attributes of each future human before the third discrete and blatant biological event of parturition and livebirth. Exposures of the gravida to multiple diverse exogenous exposures means that morphogenesis and physiogenesis of every embryo/fetus has individualized attributes for its future human lifespan. Our proposed framework based on the biological discrete events and processes spanning pre-fertilization and prenatal development, implies that personhood should be incrementally attributed, and societal protections should be graduated and applied progressively across the pre-birth timespan.

## Background

Across human history, by introducing an individual human into society, the discrete, blatant biological event of parturition and livebirth definitively marked the attribution of personhood for that individual human. In recent decades, the concept of personhood has been elaborated and debated in multiple contexts including legal, theological, secular, scientific and philosophical. Notably, discourses relating to the time at which personhood can be attributed to a developing potential human have oftentimes reached divergent conclusions (1, 2). In the book *Personhood Revisited* (3), Professor Howard W. Jones, Jr., one of the founding parents of human *in vitro* fertilization medical practice in the United States, listed the definition of personhood to be “A status acquired during human embryonic development that confers protection by society.” It is our current contention that both new and well-established scientific insights can provide a framework by which such considerations regarding development of human individuality and attribution of personhood might be better made. In the scientific realm, advances in reproductive technologies across recent decades necessitated carefully considered ethical and scientific assessments and implementation of guidelines for medical and scientific practices (4, 5). As scientific knowledge has accumulated about human birth, gestation, implantation, fertilization, gametes, and genetics, the beginning of human personhood has been defined by various endpoints related to each of these biological processes (3, 4). Associated with each such biological endpoint, ethical, legal, theological and secular social interpretations have been superimposed.

In chapter 7 entitled “*Personhood*” (3), Jones summarizes much of the history of the concept of personhood including philosophical (ethical reasoning), theological (religious beliefs), secular (includes scientific fact-based interpretations as well as other non-theological lay public opinions), and legal (laws, enforcement and court decisions) considerations regarding the concept of personhood. He makes a pragmatic comment on page 104, “As said previously, it is necessary from a practical point of view to select one of the events previously described as THE event that indicates personhood…Viability, the ability to survive without being attached to the mother is surely THE major biological milestone indicating personhood.” While we agree with his point that presumptive viability is a robust prenatal indicator of personhood, we do not agree that a single developmental stage or event may or should be pragmatically selected to fully bestow the status of personhood before birth. Our argument is that the endogenous and exogenous scientific evidence makes it clear that application of the concept of personhood must be accrued incrementally over the course of early development. It is illogical to conclude that personhood is completely absent at one moment and completely present at the next, *via* some singular instantaneous transitional event. From this fact-based and reasoning-based (scientific and philosophical) perspective, we support the contention that incremental accrual of attributes is the most coherent framework for personhood that encompasses prenatal individualization of humans by serial and concurrently acting endogenous and exogenous developmental factors.

From a philosophical perspective, the term person or personhood has been defined in numerous different ways. Some philosophers, such as John Locke, base their conception of personhood on intelligence, reason, reflection, and maintenance of a particular conscious perspective across time (6). Some, such as Immanuel Kant (7), rely on “moral agency” or the ability to perceive actions as right or wrong and take actions that can be considered moral or immoral (8). Others such as Charles Taylor say personhood is based on “matters of significance” that are “peculiarly human” such as shame, dignity and love (9). Still, others say personhood relies on free will and “reflective self-evaluation” (10), “capacity to engage in deliberation” (11), some conception of the soul (12), etc. Given these overlapping, complex, and at times contradictory conceptions of personhood, it is important that as we move forward with our framework for the biomedical basis of personhood that we clearly and sufficiently define the term for our purposes. Personhood is the idea that a living human being has a complex set of highly individualized traits, needs, and desires that make them a unique individual, with a distinct perspective of the world, and who is distinguishable from all others. To make it clear, we are asserting that without having a unique, complex mix of characteristics, a distinct perspective of the world, and being a distinguishable individual, a human cannot be said to have personhood. Additionally, our definition implicitly includes the societal protections mentioned by Professor Jones, since the main purpose “of society is to promote good and happy life for its individuals” (13). Therefore, since individual persons have distinct characteristics, needs, and desires, society must protect those with a personhood status in order to allow individuals to live good and happy lives.

As profiled by Greasley (14), there are long-standing philosophical debates about when persons begin to exist, and arguments broadly fall into either punctualist or gradualist theses. Biologically, from the earliest stages of initiation of a potential human being, accrual of progressively more advanced attributes are most rationally interpreted as being a mixture of both notable events that are limited in time but feature drastic developmental changes, but also a spectrum of processes which cannot be simplistically divided into individual events but are essential to the development of patterns of biological functions that lead to development of individual humans. In other words, biologically there are events and processes and both of these punctualistic and gradualistic elements are essential temporally-dependent components of prenatal human development.

If philosophical considerations of personhood prior to livebirth are to be rooted in observable scientific facts, then some suppositions and interpretations may necessarily change as new scientific data are gathered. For example, as regards the philosophical tractability of debate about attribution of personhood, Dworkin (15) has stated “…there is no biological fact waiting to be discovered or crushing moral analogy waiting to be invented that can dispose of the matter.” To the contrary, there are in fact recent basic laboratory research discoveries, two of which may have a profound impact on the attribution of personhood to potential humans.

First, in recent research, Palmerola et al. (16) have demonstrated that a high rate of biological attrition of preimplantation embryos in IVF is due to failure of DNA replication. It is reasonable to hypothesize that this is not merely attributable to IVF culture conditions, but rather the same process plausibly occurs in normal human reproduction. If that reasonable expectation is true in spontaneous *in vivo* fertilization events, then a punctualist view that personhood begins at fertilization would be severely compromised because that would mean perhaps 1/3 to ½ of all developing human embryos who might be designated as persons would undergo spontaneous death even before implantation.

Second, Kerepesi et al. (17) have demonstrated that individual mammalian embryos appear to have an epigenetic clock that resets to a time zero at the primitive streak stage. This is a new biological fact that has been recently discovered and arguably is a punctualistic event that should now be considered and added to the array of events and processes that are recognized to be part of early normal human development several days after implantation. Ongoing cellular and molecular research are likely to demonstrate other key events and processes in morphogenesis and physiogenesis in relevant animal models and humans.

In Table 1, we summarize correlations of biological and medical processes and events with bioethical attributions of personhood from both punctualist and gradualist perspectives. It is noteworthy that multiple important biological events and processes that occur early in human reproduction and development are highly stochastic and seem to generally be of limited interest in discussions of acquisition of personhood. For example, potential effects of germline mutagens on oocytes or spermatozoa, factors affecting gametogenesis, survivorship of preimplantation embryos, accounting for losses of potential or actual early pregnancies due to biochemical pregnancies (transient positive hCG tests, perhaps 10%–30%), spontaneous or inevitable abortions (some 15%–25%) (18) and ectopic pregnancies (about 2%) all raise objective quantitative challenges to a punctualist attribution of full personhood to zygotes or embryos around the events of fertilization, implantation and pregnancies during the early post-implantation interval. Later in pregnancy in the first and second trimesters, several of the potential obstetrical observations that may be used to document potential landmarks of progressive fetal *in utero* development require use of ultrasonic imaging which is routinely available in many locations but is not readily available in many others. Such differences raise the specter that assignment of personhood (or not) would depend upon the geographic distribution of healthcare technology. This notion that attribution of personhood to a fetus *in utero* might primarily depend upon the exogenous factor of access of the gravida to medical technology is relevant to public policy and legal status but will not be further addressed in this paper. Similarly, in many global regions, extremely preterm neonates will effectively have no plausible chance to survive due to unavailability of intensive care nursery services. Additionally, over the entire embryonic and fetal intervals, endogenous maternal and exogenous exposures influence development of diverse characteristics, many with sustained effects well into postnatal life, even lifelong.

**Table 1:** Correlations of biomedical and bioethical features of human reproduction and prenatal development.

Biomedical processes and events				Bioethical interpretations or attributions of personhood			
Process	Event	Biological remark	Medical remark	Punctualist	Associative comment	Gradualist	Associative comment
Integrity of Parental DNA	**–**	Germ cell mutagenesis and protection	–	–	Usually not assessed	–	Usually not assessed
Gametogenesis	**–**	Parental genome and epigenome packaged for possible fertilization	–	–	Usually not assessed	–	Usually not assessed
–	**Gamete attrition before and after coitus**	Multiple cohorts of oocytes per month for single ovulation and only one spermatozoan per fertilization	–	–	Usually not assessed	–	Usually not assessed
	**Fertilization**	Zygote forms; may or may not give rise to identical twins	–	Some may attribute full personhood	–	May attribute a degree of personhood	–
Pre-implantation embryo attrition	**–**	Plausibly occurs in spontaneous fertilizations, but not yet proven	High rate of demise of IVF embryos due to DNA replication errors	–	New science, not yet assessed	–	New science, not yet assessed
–	**Implantation**	Endometrium must be properly “prepared” for implantation to occur *in utero*	(a) Some are ectopic; (b) others are “biochemical” pregnancies only; (c) others become spontaneous or inevitable abortions	Some may attribute full personhood	Note that implantation of a zygote into the endometrium is the classical definition of “conception”	May attribute a modestly greater degree of personhood	–
Post-implantation embryonic development to primitive streak stage	**Epigenetic resetting of each individual's organismic clock to time zero (*t* = 0)**	Shown in animal model; stage in human is about day 15 of embryogenesis; identical twinning occurs prior to this gastrulation/primitive streak stage	IVF embryo Day 14 rule in many jurisdictions fits with these biological insights; process and events are otherwise medically discreet	Usually not addressed	Usually not addressed	May attribute some further modestly greater degree of personhood	New science about epigenetic resetting of each individual's organismic clock to time zero (*t* = 0), not yet assessed
	**Fetal heart flutter**	–	Requires ultrasound technology; so not observed in many gravidas	May be deemed adequate to attribute full personhood	–	–	Usually not assessed
	**Viable IUP on U/S at 10–12 weeks EGA**	–	Requires ultrasound technology; so not observed in many gravidas	May be deemed adequate to attribute full personhood	–	May attribute some further modestly greater degree of personhood	–
	**Quickening**	–	Range varies, but fetal movement usually felt between 16 and 24 weeks EGA	May be deemed adequate to attribute full personhood	–	May attribute some further modestly greater degree of personhood	–
	**Early second trimester morphometric OB scan**	–	Requires ultrasound technology; usually performed in contemporary OB care but not performed in many gravidas	–	Usually not assessed	–	Usually not assessed
	**Viability of extremely preterm infants with contemporary ICN care**	–	22 weeks EGA or later	Usually deemed adequate to attribute full personhood	–	Often attributes greater degree of personhood; may be deemed adequate to attribute full personhood	–
	**Viability of most infants without ICN care**	–	35–37 weeks EGA	–	Usually not assessed	Often attributes greater degree of personhood; may be deemed adequate to attribute full personhood	–
	**Term/near term**	–	Early term EGA: 37 weeks, 0 days to 38 weeks, 6 days. Full term EGA: 39 weeks, 0 days to 40 weeks, 6 days.	Usually deemed adequate to attribute full personhood	–	Usually deemed adequate to attribute full personhood	–
	**Livebirth of neonate**	–	Births: May be spontaneous, augmented, or operative	Is certainly deemed adequate to attribute full personhood	–	Is certainly deemed adequate to attribute full personhood	–
	**Postnatal life**	Continued growth and differentiation subject to multiple endogenous and exogenous factors	Growth charts, developmental landmarks	–	–	–	Continued acquisition of attributes of personhood

## Prenatal determinative transformational events

Many genetic, epigenetic, and developmental *milieu* effects from pre-fertilization through livebirth determine each individual's unique combination of attributes upon societal entry. For our purposes in this paper, we define two terms as follows:

1. Potential human: The stages of human development spanning initial interactions of one female and one male gamete such as the spermatozoa binding to the zona pelucida up to gastrulation, formation of the primitive streak, resetting of the epigenetic clock, progression through gestation and parturition.
2. Physiogenesis: The partner concept to morphogenesis; meaning the acquisition of molecular, cellular, tissue, organ, system and organismic level functions to allow future individual life. Morphogenesis and physiogenesis determine future potentialities for individual persons.

We contend that there are three discrete events and multiple continual/continuous processes that determine the individual attributes of a potential human. We present our framework that during prenatal development, a potential human attains a personhood status through the overlaying of a complex individualization process onto the three known discrete determinative events, namely fertilization, the novel epigenetic time reset and livebirth. The individualization process during prenatal development is the product of a spectrum of factors, both endogenous and exogenous, that incrementally influences acquisition of multiple attributes and thus results in an incremental accrual of personhood prior to and then fully upon livebirth.

Although we argue for the importance of the recently discovered biological event of resetting of the epigenetic clock (approximately Day 15 of embryonic life for human embryos) (17), we also argue that it is insufficient to solely determine personhood because no single developmental event adequately encompasses the entire process of prenatal individualization of humans. Across the course of prenatal development, becoming an individual depends upon multiple genetic, epigenetic and other morphogenic/physiogenic organizational processes such that individualization is a summation of all of the developmental determinants that cause or modify the acquisition of biologically definable metabolic, morphologic, behavioral, and reproductive attributes.

From a biomedical point of view, characterizing the pre-birth developmental course of human beings as a parsing of observable facts into two presumptively distinct categories of events versus processes is admittedly somewhat simplistic, but does serve as a useful construct in research, organization and documentation of scientific knowledge, and in medical practice. We shall use this bipartite approach to (a) review the various events and processes that are known to be relevant to pre-birth development of humans and then to (b) argue that to the extent that biomedical facts underlie the progressive acquisition of attributes that are relevant to life *per se*, health, longevity and functional status (potential quality of life parameters), an incremental rather than absolutist attribution of personhood is more parsimonious with biological facts.

## The two discrete unseen events of fertilization and epigenetic time reset

### Fertilization and parental genetics

It is widely understood that the genetic attributes of each individual are robustly dependent upon parental genomes and that individual diversity within a population is profoundly dependent upon random assortment of alleles and meiotic recombination that had occurred in parental gametogenesis. The key biological consequence of these processes in gametogenesis is diversification of the genomes of progeny. Each array of inherited genes provides combinations that may mimic or diverge from the phenotypic traits of the parents, because most human traits are a product of a non-Mendelian complex interaction of multiple genes (19).

### The epigenetic organismic clock reset to time zero (*t* = 0)

The broad consensus regarding epigenetics in human embryonic development is that there is a general “wiping the slate clean” but also a modest degree of retention of some parental epigenetic marks (20). This predominant but incomplete erasure of epigenetic marks has attracted great research interest as well as debate about how important or unimportant epigenetic inheritance may be for any individual (21–26).

As an outgrowth of epigenetics research in the embryo, Kerepesi et al. (17) have studied the temporality of epigenetic events. Their recent publication makes the case that they have characterized the time and event that is the beginning of aging for each individual mammal. These investigators used machine learning to develop a new multi-tissue epigenetic clock. They used this new clock and others to assess aging in prenatal mammalian development. In the mouse model, these investigators reported a rejuvenation period during early embryogenesis and the onset of the beginning of aging after this rejuvenation event. Their observed epigenetic age minimum in the mouse was at E6.5/E7.5 which corresponds to gastrulation, formation of the primitive streak and the three germ layers. This embryonic developmental stage in human embryos occurs on approximately embryonic day 15. Kerepeski et al. (17) go on to state the following:

Our study suggests that the germ line ages but is rejuvenated in the offspring at some point during early embryogenesis. This rejuvenation occurs during early post-implantation stages corresponding to gastrulation when the offspring reaches its minimal biological age. We propose that this minimum, the ground zero, marks the beginning of aging of an organism… The data indicate that ground zero lies between E4.5 and E10.5 in mice, most probably at E6.5/E7.5. This period corresponds to the germ-layer specification (gastrulation) accompanied by the exit from pluripotency… Our current work now pinpoints the beginning of aging to ground zero… Overall, this work identifies a natural rejuvenation event during early life and suggests that organismal aging begins during embryogenesis, approximately at the time of gastrulation.

## Diverse exogenous factors influence pre-fertilization, embryonic and fetal attributes of individual humans

The range of potential cell types during human *in utero* development that might be affected by exogenous factors are not yet fully elucidated, but there is a global effort underway to do precisely that; namely, to create a comprehensive reference map of cells during development (27). Even though the precise identity of cellular targets during development are not fully known, stochastic exogenous environmental and intrauterine *milieu* factors are known to impact the developmental trajectory of humans (21, 28). The conceptual challenge in understanding prenatal development with or without exogenous perturbations is to formalize how emergent properties arise *via* dynamic interactions at both higher and lower levels of biological organization. Such theoretical and analytical network modeling research is occurring (29), with, for example, more than 100 studies currently underway under the auspices of the National Institutes of Health MultiScale Modeling Consortium (https://www.nibib.nih.gov/research-funding/interagency-modeling-and-analysis-group-imag).

Disruptions of structural and functional development occur and there is a long history of scientific study of such variations from the normative patterns. Specifically, the scientific discipline of teratology was historically rooted in morphogenesis and disorders thereof, and we assert that the later broader term of developmental toxicology has helped add dynamic integrated elements that we herein call “physiogenesis” as its *sine qua nom*, as it focuses on how exposures to various physical, chemical, biological and social factors may adversely affect the health and functional well-being of an individual human.

**Figure 1:**
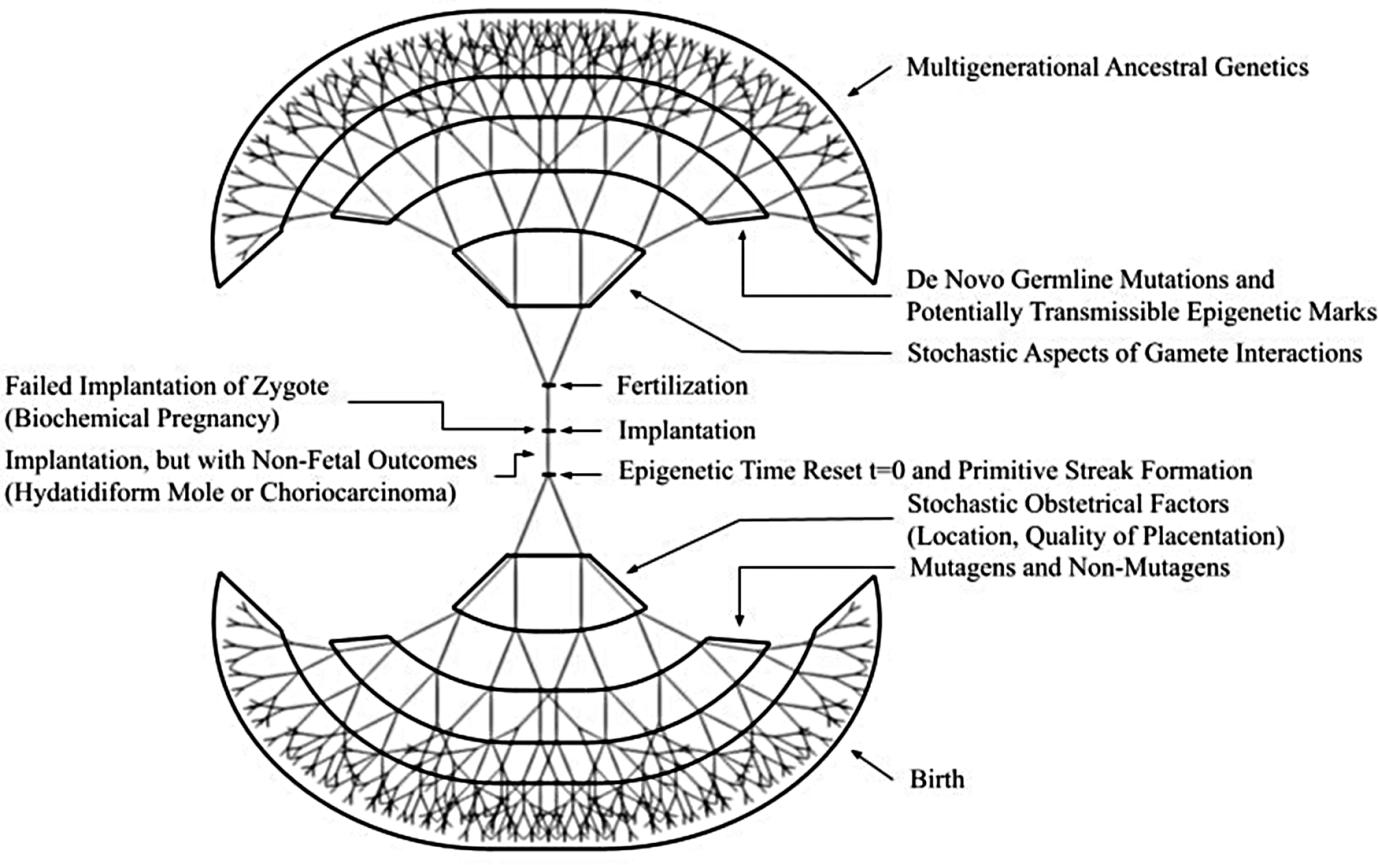
Framework for sources of diversity for developmental individualization of potential and future humans. Both pre-fertilization (the upright tree) and post-fertilization (the shared trunk and inverted tree) elements illustrate the complexity* of endogenous and exogenous events and processes by which attributes of personhood are incrementally acquired. The fractal-like elements are intended to be evocative in that future non-deterministic mathematical modeling could be undertaken. *The items in the figure are representative, not all-inclusive.

While teratology/developmental toxicology research primarily seeks to understand adverse effects of the many exposures that inevitably occur during development of an individual human, it is widely acknowledged that many exposures will either have healthful effects or have no plausible impact on the future well-being of that individual human. Thus, we herein offer the inverse view that taken *in toto*, whether adverse, salutary or neutral, the effects of diverse exogenous exposures alter the developmental trajectories of each future individual human and consequently provide countless patterns of individualization of all future humans (Figure 1). In Figure 1, we have elected to illustrate our framework of pre-birth human individualization during development as a fractal drawing. We suggest that given the applicability of fractal models to multiple biological systems such as cardiac function (30), the lung (31), and even the entirety of life itself (32), that the human individualization framework would be a good subject for application of fractal mathematics in non-deterministic modeling.

After implantation, the fetal environment and the fetal developmental trajectory is understood to be dependent upon maternal metabolism (nutritional adequacy, excesses or inadequacies), intentional exposures (dietary choices, personal habits, substance use or abuse, etc.), environmental/unintentional exposures (physical, chemical or infectious agents and familial and socioeconomic stressors), as well as maternal pre-pregnancy comorbid diseases and obstetrical disorders such as gestational diabetes, preterm labor, and early or late onset preeclampsia. All of these factors (33) (see multi-authored chapters 3–10) may influence fetal development and thereby move the fetus along different developmental paths than is followed by others. Once again, each of these types of exposure during development modify that future human and diversifies the spectrum of persons who later enter society *via* parturition and livebirth.

There are a number of classes of exposures that are well-known to affect human development prior to livebirth. Some of these prenatal exposures are quite specific such as drugs prescribed to pregnant women to treat an underlying disease (34, 35); while others are environmental such as inhaled particulates that appear to adversely affect infants’ respiratory and immune systems, brain development, and cardiometabolic health (36). Additionally, there are many exposures that are not proven with certainty to affect human prenatal development but are widely thought to be probable or possible human teratogens/developmental toxicants. The following are brief profiles of the key classes of such developmentally influential agents.

### Mutagens

All humans are exposed to mutagens (37) including oxidative stress, ionizing radiation, naturally occurring compounds such as mycotoxins as well as environmental contaminants and pharmaceutical agents. Exposures to mutagens and consequent stochastic mutagenic effects (38) during the stages of parental gametes, zygote, embryo and fetal intervals are a source of *de novo* mutations that are acquired differences in an individual's genome. These individualization effects are dependent upon the several characteristics of the exposure such as the compound or compounds, the dose, the duration, the timing during developmentally sensitive windows, and the individual's or maternal genetics that may modify resistance or susceptibility to the effects of mutagens in various tissues and organ systems.

Prior to fertilization, the individuality of a potential human derives from the genetic diversity among the oocytes and spermatozoa that were produced by the potential parents, including acquired germline mutations. Obviously, the genetics of the potential mother and father are profoundly important in determining the spectrum of individual possibilities for that potential human. Another source of individuality can derive from the age of the parents, particularly the father. The reasoning is that the prolonged decades of sperm production offer more opportunities for *de novo* mutations to occur and be present in spermatozoa that may participate in fertilization of an oocyte (25).

### Non-mutagens

Humans are exposed to many bioactive chemicals which do not exert effects primarily by causing mutations. Some non-mutagenic chemical exposures are intentional, such as use of medicinal drugs or substances that are recreational or addictive that may be legal or illicit. Maternal exposures to these classes of compounds during pregnancy are known in many instances to produce adverse effects on the embryo or fetus. Less clear-cut but potentially important are exposures to these classes of bioactive chemicals of either parent prior to or during gametogenesis, fertilization, implantation and embryonic stages of development even prior to awareness of pregnancy.

Since it is widely reported that only about one half of all pregnancies are planned, a vast majority of women take at least one prescription drug during pregnancy, and approximately two thirds take a medicinal drug of some sort during the first trimester. There are numerous pharmaceutical drugs that have been reported to cause birth defects when exposures occurred *in utero* (35, 39). Even for common over-the-counter medications such as those containing acetaminophen (paracetamol), there is justification for considering that fetal developmental effects on neurological, reproductive and urogenital systems may occur (40). Some uncertainty about associating prescription or over-the-counter medication use with developmental effects is valid because the mother typically has an active disease or undiagnosed signs and symptoms, any of which may present certain risks *per se* to the fetus; then the usage of a drug is superimposed in that setting. Nonetheless, regulatory authorities require manufacturers to report such adverse events through safety reporting systems and such data demonstrate to some extent the potentialities for effects in the offspring.

The other prominent class of voluntary chemical exposures that could impact development *in utero* is recreational or addictive substances. As noted currently by the U.S. National Institute on Drug Abuse (41), these include marijuana (cannabis), stimulants (cocaine and methamphetamine), MDMA (3,4-Methylenedioxymethamphetamine, Ecstasy, Molly), opioids (heroin, diverted prescription opiates), alcohol (ethanol) and nicotine (tobacco products and e-cigarettes). The scientific literature demonstrating the impact of these classes of exposures and the broad range of developmental effects is truly massive (41) and unequivocal.

Less certain but of potential significance is the suggestive evidence from a number of research reports showing that exposures that precede the fetal interval may pose an explicit morphogenic developmental risk or have a physiogenic effect that only manifests in the offspring long after livebirth. We shall cite one of each for illustration.

First, in a recent meta-analysis, Zhang et al. (42) studied the relationship between parental alcohol exposure and risk of congenital heart disease (CHD) in their offspring. In the pooling of data from more than 50 studies with >40,000 CHD cases and >290,000 controls, there was the expected finding of an increased risk of CHD with maternal alcohol exposures [odds ratio (OR) = 1.16; 95% confidence interval (CI): 1.05–1.27] but also with paternal (OR = 1.44; 95% CI: 1.19–1.74) alcohol exposures. To the best of our knowledge, the molecular or cellular mechanisms underlying this reported risk are unknown.

Second, Northstone et al. (43) used the Avon Longitudinal Study of Parents and Children's questionnaire data on smoking and smoking onset from >9,800 fathers and the data regarding growth of their children from 7 to 17 years. In brief, after adjusting for potential confounders, the investigators found that for the men who reported regular smoking at <11 years of age, the adjusted mean differences in BMI, waist circumference and total fat mass in their sons were significantly greater from age 13 onwards. From the concept that smoking by boys in mid childhood may contribute to obesity in adolescent boys of the next generation (43), Hammer et al. (44) recently reported data from their work in an animal (mouse) model, offering a degree of mechanistic explanation for non-genomic transmission of such observations about paternal physiogenic effects on their offspring as follows: “miRNAs in the plasma microenvironment of spermatozoa may represent a mechanism for transmittable epigenetic changes to offspring and development of metabolic or respiratory diseases, further highlighting paternal smoking as potential risk factor for offspring's health.” These data strengthen the idea that tobacco smoking by human male teenagers increases the risk of overweight and obesity in their male offspring.

### Parental diseases

Numerous chronic or recurrent diseases occur in women who become pregnant, sustain the pregnancy, and undergo labor and delivery. The range of such concurrent illnesses that could potentially impact growth and development of each future human was demonstrated by Jolving et al. (45) in their nationwide (Denmark) registry-based cohort study that included all women who gave birth (>1.3 million) between 1989 and 2013. These investigators specified 23 maternal chronic diseases within the decade preceding childbirth. While noting that the overall prevalence of maternal chronic disease increased from 3.71% in 1989 to 15.76% in 2013, the ten most prevalent diseases during pregnancy were chronic lung disease/asthma (1.73%), thyroid disorders (1.50%), anxiety and personality disorders (1.33%), mood disorders (0.74%), epilepsy (0.69%), inflammatory bowel diseases (0.67%), polycystic ovarian syndrome (0.52%), diabetes mellitus (0.48%), hypertension (0.43%) and rheumatoid arthritis (0.38%).

While much remains unknown about the potential impacts of many such comorbid conditions on a fetus during gestation, several maternal diseases that frequently occur during pregnancy are known to impact fetal development, ofttimes posing risks to normal *in utero* growth and development. To the extent that potential fetal developmental effects of medications used to treat such diseases can be assessed and set aside, there are still clear attributable risks to the fetus if the gravida has one or more comorbid diseases. Such demonstrated risks include fetal growth (small for gestational age/low birth-weights or high birth-weights) but also metabolic and neurocognitive effects in the neonate and the child. For example, associations of maternal comorbidities during pregnancy and perinatal and childhood outcomes include (39, 46) the following:

– Maternal depression with low birth-weight infant and later central adiposity in the child;
– Maternal diabetes (type 1, type 2 or gestational), hyperglycemia or obesity with high birth-weight infant and later metabolic syndrome and obesity in the child;
– Maternal overweight/obesity with higher likelihood of autism spectrum disorders or neurodevelopmental delays;
– Maternal asthma (with exacerbations) with increased risk of preterm delivery and low birth-weight infant (particularly in males);
– Maternal sleep deprivation and sleep-related breathing disorders with low birth-weight infant (small for gestational age) and a higher risk of mortality;
– Maternal anemia with low birth-weight infant (intrauterine growth retardation) and increased risk of preterm delivery; and
– Maternal hypertensive disorders of pregnancy (gestational and chronic hypertension, preeclampsia) with higher likelihood of childhood mental disorders.

While it may seem intuitive that maternal diseases during pregnancy could impact outcomes of her infant, some data show that paternal health also influences the health of his infant. It is reasonable to suppose that the father's health could be a heritable precursor of sorts for his infant or that cohabitation of the two parents might lead to predisposing environmental effects on their offspring. However, at least some interesting correlations that may be causations were suggested by Kasman et al. (47) in their inquiry about whether prepartum and neonatal outcomes are associated with pre-existing paternal health factors. In this retrospective cohort study in the United States of children born between 2009 and 2016, paternal health status as reflected in diagnoses of various chronic diseases was compared to the primary outcome of preterm birth (meaning live birth before 37 weeks), as well as several secondary outcomes including low birth-weight, neonatal intensive care unit (NICU) stay, gestational diabetes, preeclampsia, eclampsia, and length of maternal stay. By use of a research database covering reimbursed health care claims data on inpatient and outpatient encounters through employment-sponsored health insurance, the investigators assessed 785,809 singleton live births, with 6.6% born preterm.

Kasman et al. (47) reported, “The presence of paternal comorbidities was associated with higher odds of preterm birth, low birth-weight (LBW), and NICU stay. After adjusting for maternal factors, fathers with most or all components of the metabolic syndrome had 19% higher odds of having a child born preterm (95% CI 1.11–1.28), 23% higher odds of LBW (95% CI 1.01–1.51), and 28% higher odds of NICU stay (95% CI 1.08–1.52). Maternal morbidity (e.g., gestational diabetes or preeclampsia) was also positively associated with preconception paternal health.” These findings suggest but do not causally establish that preconception paternal comorbidities may modestly influence obstetrical and neonatal outcomes.

In addition to those parental disease and drugs exposures, each potential human is subject to numerous other chemical, social, stochastic obstetrical and microbiological exposures that are known or suspected to impact human embryonic/fetal development. It is not possible to fully review all of the possible embryonic/fetal exposures in this document, but we will describe a few notable classes.

### Environmental chemicals

There are hundreds of naturally-occurring and man-made compounds that have been demonstrated to be present in either amniotic fluid (48–51) or umbilical cord blood (52), raising the reasonable prospect that at least some of them may influence development. One prominent group of compounds has been named “endocrine-disrupting chemicals” (EDCs) which means “an exogenous chemical, or mixture of chemicals, that can interfere with any aspect of hormone action” (53). There is now broad agreement but not consensus that “Individuals and populations are exposed to EDCs, and common non-communicable diseases have been associated with environmentally-relevant doses of EDCs in human epidemiological studies…It is now well established that developmental exposure to EDCs can alter the epigenome of offspring, affecting gene expression and organogenesis, thereby altering an organism's sensitivity to disease later in life” (53). More recent research into the potential neurodevelopmental effects of EDCs (54), suggest that some effects may include alterations in brain development that advance or delay puberty or alter neuroendocrine control of reproduction thus impairing fertility. Various data suggest that neurodevelopmental effects of EDCs may occur *via* action on steroidal and non-steroidal receptors but also *via* alterations in enzymatic, metabolic and epigenetic and other cellular pathways during development (54). It may be possible to link such mechanistic complexity of EDC actions to various individual neuropsychiatric outcomes by use of network analysis tools. In a recent paper, Raja et al. (55) report that such an analysis seems to show that genes, receptors and signaling pathways interact as a consequence of exposures to EDCs and in turn are associated with disorders such as major depression, alcoholism, psychotic disorders, autism spectrum disorder, attention-deficit/hyperactivity disorder (ADHD), cerebral palsy, and sex-specific aggressive and emotional behavior.

### Other environmental stressors

Maternal environmental stressors (natural disasters; famine, social stressors) also influence development of her offspring. Tragic large-scale disasters have served as natural (or man-made) experiments wherein many pregnant women were exposed to various forms of deprivation or other stressors, and subsequent assessments of their offspring have shown a number of important health effects.

One such well-recognized event was the Dutch famine of 1944–1945. Even now, decades later, additional reports are being published that demonstrate the life-long persistence of the *in utero* developmental effect(s) that occurred. Lumey et al. (56) analyzed the heights and weights of 371,100 men in the Netherlands when they were examined for military service at age 19 years. The men born between 1943 and 1947 either did or did not experience prenatal exposure to the Dutch famine. These investigators found that there was an overall 1.3-fold increase in the risk of being overweight or obese at age 19 after prenatal famine exposure in early gestation, and an attendant 30% increase in overall mortality through age 63 relative to those with a normal BMI.

A second such large-scale disaster was the 1998 Quebec Ice Storm. Within the last few years, Paxman et al. (57) sought to gain some mechanistic insights into the physiological phenotypes seen in the population exposed *in utero* by use of proton nuclear magnetic resonance spectroscopy to analyze urinary metabolomes of male and female adolescents. Overall, these investigators found that higher prenatal stress exposure led to alterations in metabolic pathways involved in energy metabolism and protein biosynthesis; findings that are consistent with dysregulation as would be expected in insulin resistance, diabetes, and obesity.

Finally, a third such event was the Great Tangshan Earthquake (China) in 1976. In this instance, another recent study by Guo et al. (58) assessed data from >94,000 Chinese individuals born between 1975 and 1979. The investigators studied the relationship between the occurrence of schizophrenia (diagnosed by psychiatrists) and earthquake severity by seismic intensity. In brief, in comparison to an unexposed cohort, the cohort exposed to the earthquake *in utero* had higher risk of schizophrenia (odds ratio, 3.38; 95% CI 1.43–8.00). Notably, earthquake exposure during the first trimester of pregnancy showed a further increased risk of adulthood schizophrenia (odds ratio, 7.45; 95% CI 2.83–19.59).

Though such disasters illustrate the impact that external factors may have on development, societal elements that broadly affect many more individual future humans are embedded in the concept of socio-economic status (SES) as a summary of the availability of material and social resources to any individual. Childhood SES is one of the strongest predictors of lifelong well-being and appears to be associated with the duration and functional complexity of individual brain development (59).

The cellular and molecular mechanisms that mediate such effects of SES or other social factors such as familial adversity are not fully understood, but epigenetics appear to play an important role (60, 61). As noted by Tremblay et al. (60);

…there is now emerging evidence that early social-familial adversity leads to long lasting epigenetic alterations. These alterations may influence brain development, and, consequently, the ability to learn to regulate and control aggressive behaviour…the finding that many of these biological factors involved in chronic physical aggression develop very early (i.e., before birth), highlights the need to not only study the impact of the early postnatal environment on physical aggression, but also to take into account what is happening between conception and birth.

### Stochastic obstetrical factors

Other variables that impact development of some individual future humans are stochastic obstetrical factors. There are occasional presumably random events related to location and qualitative aspects of implantation and thus placentation *per se*. For example, if random implantation occurs over an underlying uterine leiomyoma (fibroid), then as the pregnancy progresses, that fetus may be subject to reduced placental perfusion or placental abruption events that could impact growth *in utero*, lead to premature delivery or pose a risk of hemorrhagic compromise or even stillbirth. Another variable that may be random or iatrogenic as part of infertility therapies is twinning, or higher multiple birth. This latter random pre-birth impact on the potentialities of one identical twin versus the other, has been convincingly demonstrated by Groene et al. (62). These investigators have recently reported on their study of monochorionic diamniotic twins (MCDA) with selective fetal growth restriction (sFGR). They found that “In MCDA twins with sFGR, the smaller twin presents with a lower intelligence quotient across all indexes and an increased rate of mild NDI [neurodevelopmental impairment] compared with the larger co-twin. To our knowledge, we are the first to show that FGR poses a substantial risk for long-term neurodevelopment in this unique identical twin model controlling for maternal, obstetrical, and genetic factors.”

### Maternal and fetal microbiomes

New scientific discoveries demonstrate the impact of the maternal microbiome (63) and present a current controversy about the presence and potential effects of a fetal microbiome on prenatal development and perinatal outcomes. The key points are that the maternal vaginal microbiome influences the risk of preterm birth (64) and there is evidence for and arguments against the presence of a fetal microbiome (65–67). Some evidence supports the notion that the long-held “sterile womb” dogma may be fading as evidence for an *in utero* colonization hypothesis has become more substantiated (66). Some additional data suggest that fetal immunological development *in utero* at least in part depends upon prepartum exposure of each fetus to microbes as early as the second trimester; from Mishra et al. (65):

The events occurring during fetal gestation are essential for the overall development and growth of the individual…Various studies have recently suggested that certain antigens as well as bacterial entities may cross the placental barrier and make their way to fetal organs…these findings have wider implications in understanding the key factors involved in fetal immune system development and priming *in utero*, which may set the basis for life-long health and immunity of the organism…the existence of a spatially diverse microbial signal in fetal tissues, their ability to culture-expand anaerobically, and the presence of microbial antigen-specific memory T cell activation, are difficult to reconcile with either systematic biases or random noise. Collectively, our data suggest a low but consistent presence of microbes in at least some of the healthy human fetuses in the 2nd trimester of gestation.

Nevertheless, a recent multidisciplinary argument by Kennedy et al. (67) makes the opposing case saying that:

Here we evaluate recent studies that characterized microbial populations in human fetuses from the perspectives of reproductive biology, microbial ecology, bioinformatics, immunology, clinical microbiology and gnotobiology, and assess possible mechanisms by which the fetus might interact with microorganisms. Our analysis indicates that the detected microbial signals are likely the result of contamination during the clinical procedures to obtain fetal samples or during DNA extraction and DNA sequencing…The pursuit of a fetal microbiome serves as a cautionary example of the challenges of sequence-based microbiome studies when biomass is low or absent, and emphasizes the need for a trans-disciplinary approach that goes beyond contamination controls by also incorporating biological, ecological and mechanistic concepts.

While the fetal microbiome concept remains unsettled, there is wide acceptance in obstetrics that some vaginal organisms contribute to ascending infections that produce chorioamnionitis and preterm labor and deliveries. Recent data also show that even in the absence of intrauterine infections, the maternal vaginal microbiome influences the risk of preterm birth (64). In their study of spontaneous preterm birth, Flaviani et al. (64) studied the interactions between the cervicovaginal metabolic environment and microbiota in tandem with the host innate immune response in a prospective United Kingdom (UK) longitudinal cohort of pregnant women. Analysis of cervicovaginal samples (10–15 + 6 weeks) identified potentially novel interactions between risk of spontaneous preterm birth (sPTB) and microbiota, metabolite, and maternal host defense molecules in an ethnically heterogeneous pregnant population (*n* = 346 women; *n* = 60 sPTB < 37 weeks’ gestation, including *n* = 27 sPTB < 34 weeks). These findings indicate that the maternal microbiome may poise a pregnancy to either be more likely to go to term, or to deliver prematurely and thereby impact an individual neonate and modify its post-natal developmental trajectory and ofttimes compromise one or more of its life-long functional outcomes.

All of these sources of developmental diversification of individual humans that span the time and events from maternal and paternal gametogenesis across pre-fertilization, fertilization, implantation and embryonic and fetal development up to parturition, provide the biomedical background of processes and events that largely determine the characteristics and potentialities of each person at the time of birth.

## The third discrete and blatant event of parturition and birth

The event that introduces a human into society is parturition and livebirth, and this process presents its own set of challenges that may make a penultimate impact on the developmental outcome of the neonate. For the fetus, several maternal medical and obstetrical risk factors pose risks that may influence the infant's lifelong health and functional status in diverse ways.

In High Income Countries (HIC), some of the common maternal factors at the time of parturition that impact the neonate are hypertensive disorders of pregnancy (including preeclampsia), age, weight status, diabetes (pre-existing or gestational), substance use, depression, breech presentation and previous Cesarean birth(s) (68). Among the most dangerous obstetrical complications during labor and delivery for the fetus are uterine rupture, shoulder dystocia, umbilical cord prolapse, chorioamnionitis and fetal macrosomia.

In Low and Middle Income Countries (LMIC), the recent study by Baguiya et al. (69) show the devastating impact of maternal pre-existing conditions and suspected or confirmed infections on neonatal outcomes. This multinational team of investigators conducted a study in 2017 in 408 hospitals in 43 LMIC in all WHO regions. Women (*n* = 1,219) with suspected or confirmed infection during pregnancy at 28 weeks or more of gestational age were followed, along with their infants, up to day 7 postpartum. Neonatal Near Miss (NNM) cases were defined by the criteria of birth-weight <1,750 g, gestational age at birth between 28 and 33 weeks, 5 min APGAR score <7, or use of any of several acute interventions (e.g., parenteral antibiotics, ventilation support, Intubation at birth or other medical or surgical interventions). These investigators reported neonatal outcomes to be

1) 64% (*n* = 780) babies alive without severe complications,
2) 25.9% (*n* = 316) were NNM cases and
3) 10.1% (*n* = 123) perinatal death (stillbirth and early neonatal death);

and commented “Overall, one-third of births were adverse perinatal outcomes. Pre-existing maternal medical conditions and severe infection-related maternal outcomes were the main risk factors of adverse perinatal outcomes.”

In multiple ways, whether births occur in HIC or LMIC, any of these peripartum risks may impact the fetus/neonate by causing cerebral palsy, Erb's palsy, hypoxic ischemic encephalopathy (“HIE”) (perinatal asphyxia), cerebral hemorrhages, hematomas or periventricular leukomalacia (“PVL”) as seen in premature infants. If PVL (which can lead to cerebral palsy or epilepsy) is seen as an overt consequence of premature birth, there is strong evidence of more subtle central nervous system developmental effects as well, since a recent study in a cohort of >4 million persons found that preterm and early term birth were associated with significantly increased risks of autism in boys and girls (70).

In summary, all of these antecedent diverse, mostly adverse, effects change the life-long trajectories of countless humans in multiple ways prior to birth. Humans are individualized by endogenous genetic and epigenetic events but also by effects of and responses to exogenous exposures within this developmentally sensitive interval. Developmental exposures of potential humans to multiple diverse exogenous factors means that morphogenesis and physiogenesis of every embryo/fetus possesses individualized attributes for its future lifespan.

## How do medical and scientific research guidances relate to our scientific and ethical framework of prepartum human development?

In historical terms, as human *in vitro* fertilization procedures were developed and procedures were regularized in this area of medicine, reviews were conducted by multi-disciplinary committees that provided insightful considerations from many diverse perspectives. Two leading examples were those published in the United States (4) and the United Kingdom (5). While the US panel's report provided careful evaluative remarks on “The Status of the Early Human Embryo” (4), the UK panel's report, widely called “The Warnock Report” also made careful evaluative remarks (5) and proposed that human embryos should not be sustained *in vitro* for more than 14 days for any purpose. In some jurisdictions (such as the UK and Australia), this proposal became law (71), and in virtually all portions of the globe, this interval became the explicit or *de facto* guidance for all clinics and laboratories that participated in human *in vitro* fertilization clinical practice and/or research. In the book *Personhood Revisited* (3), Jones reviewed much of the history of *in vitro* fertilization as its practices developed in the UK, US and other nations. Regarding personhood, Jones commented that the American Fertility Society Ethics Committee in 1986 agreed that personhood was the status which was acquired during development at a time when protection by society was expected. Also as defined, this equated to the theological concept of ensoulment in regard to the requirement of societal protection. The Committee also considered the basic biological fact that over the first several days after fertilization, it is uncertain how the development of that entity will progress. Outcomes may be an individual human, multiple individual humans (identical twinning), a benign hydatitiform mole tumor or a malignant chorioepithelioma tumor. The committee's assessment was “There was general agreement that the earliest possible point in time for the acquisition of personhood—i.e., protection by society—occurred with the appearance of the primitive streak (The President's Council on Bioethics 2002), which itself guaranteed biological individualization and eliminated the possibility of a benign or malignant tumor” (3, 72). This led to the recommendation that 14 days would be the appropriate designated interval.

Over the subsequent decades and particularly in recent years (71) there have been countless thoughtful discussions about the “14-day rule;” however, only recently has a pertinent professional scientific society taken the step of formally changing their position. Specifically, in May 2021 the International Society for Stem Cell Research (73), issued their “Guidelines for Stem Cell Research and Clinical Translation” and included on page 12 their argument for changing and support for ending the 14 days post-fertilization restriction. As expected, this new position has evoked great interest and commentary among many medical and scientific professionals (74–76). One such commentary by Mummery and Anthony (77) noted that considerations are being given to diversifying new guidelines that will hopefully be “fit for purpose,” so that the opportunities for scientific discovery are pursued within an explicit and transparent ethical framework. We hope that no matter the perspective of any person or the position of any organization engaged in this renewed discourse about the “14-day rule,” each will duly consider the framework we are presenting herein as they formulate their new or renewed points-of-view. Our proposed framework as profiled in Table 1 and illustrated in Figure 1, is based on the biological facts spanning pre-fertilization and prenatal developmental discrete events and processes and implies that personhood should be incrementally attributed and societal protections for potential humans should be graduated and applied progressively across the entire human developmental pre-birth timespan. However, the specific structure of these societal protections is outside our area of expertise and thus beyond our purview. Therefore, we will not attempt to propose any laws or policies in this paper.

## Summary and conclusions

Personhood is a designation assigned by living humans to living humans and is the idea that a living human being has a complex set of highly individualized traits, needs, and desires that make them a unique individual with a distinct perspective of the world, and who is distinguishable from all others. In the biomedical context, a living human being can be reasonably well defined, understood and characterized while the superimposed designation of “person” cannot be fully profiled in biological terms. Nonetheless, setting aside some valid discussions about whether members of another species might merit designation as “a person,” it is implausible for there to be “a person” in the absence of a living human being. Thus, biological bases are necessarily foundational for developing any construct of when a human being might be deservedly designated as a person. It seems unlikely that many reasoning persons would argue that upon birth, a living infant human being is not a person. The endless debates obviously relate to the criteria or the criterion by which personhood is or might be designated in the pre-birth interval for a developing human.

Much careful thought and debate has been invested in considering whether there is some discrete event or moment that defines without equivocation that point in a developmental trajectory when personhood might be fully assigned. Running somewhat in parallel to that discourse, the concept of more continuous accrual of personhood during pre-birth development cumulatively leading to full personhood, has also been developed and argued.

We argue that it is an ethical imperative to consider more than merely selecting or endlessly debating when a human is also a person. It is also incumbent upon us to include fundamental considerations of the future human potentialities of individual persons that encompass both the duration of life (longevity) and the quality of life, such as health-related quality of life, over that individual's future lifespan. It seems likely that most persons would want as many newborns as possible to have favorable future potentialities by not being born with or at elevated risks of congenital cytomegalovirus (CMV), *in utero* Zika virus infection, extreme prematurity and its associated risks, fetal alcohol syndrome or exposures to other drugs of abuse *in utero*, major congenital anomalies (cleft palate, spina bifida, others), cerebral palsy, several types of developmental delays in children (cognitive, motor, social, emotional, behavioral or speech delays), predisposal to early-in-life onset of metabolic disorders such as childhood hypertension or other cardiovascular diseases, type 2 diabetes, kidney disease, frailty or obesity, gastrointestinal diseases, immunological disorders, cancers, etc. Myriad variations among persons are a key part of the way in which society is enriched by diversity. At the same time, it is not a eugenics consideration to strive to have the largest number of neonates to have undergone a pre-birth developmental trajectory that offers breadth and depth of future potentialities later in life.

As modern scientific discoveries have revealed demonstrable facts about additional endogenous and exogenous biological processes and events prior to birth, scientific and ethical considerations are pertinent to assess any other discrete or continuing components that contribute to individualization before the societal entry of an individual human by livebirth. We present the endogenous genetic and epigenetic events and exogenous developmental *milieu* processes that, when combined, produce the distinguishing features of a human prior to its entry into society *via* livebirth. Accordingly, we present the novel argument that there are now not one but two known discrete unseen biological events that are pertinent to determining a future individual's identity; namely, fertilization plus the additional discrete biological event of epigenetic resetting of that individual's biological organismic clock to time zero (*t* = 0) at the gastrulation stage (around day 15 of embryogenesis). Additionally, those two discrete unseen biological events are immersed in a continual complex developmental process that spans pre-fertilization and gestational intervals and drives individualization of diverse attributes of each future individual human, preceding the discrete and blatant biological event of livebirth and societal entry of that human. During prenatal development, each future human is subject to various and variable exposures of the gravida to multiple physical, chemical, biological and social factors as well as to its own unique maternal *in utero* environment. These diverse exogenous exposures of every embryo/fetus influence its morphogenesis and physiogenesis thereby individualizing its future attributes, health and functional well-being across its individual human lifespan within society. However, it is important to note that attribution of personhood to a developing human at or before birth is not entirely an end unto itself. Even following early development and birth, the attributes that define an individual's personhood continue to change. From the perspective of an obstetrician or pediatrician, initiation of a new individual human life is only the beginning of what is a person's life trajectory that has hopefully been developed as a consequence of favorable pre-birth factors.
